# Parental perspectives on negotiations over diet and physical activity: how do we involve parents in adolescent health interventions?

**DOI:** 10.1017/S1368980021000458

**Published:** 2021-06

**Authors:** Sarah Shaw, Sara Correia Simao, Sarah Jenner, Wendy T Lawrence, Kathryn Woods-Townsend, Christina A Vogel, David Farrell, Hazel Inskip, Janis Baird, Leanne Morrison, Mary Barker, Sofia T Strömmer, the EACH-B study group

**Affiliations:** 1 MRC Lifecourse Epidemiology Unit, University of Southampton, Southampton General Hospital, Southampton SO16 6YD, UK; 2 NIHR Southampton Biomedical Research Centre, University of Southampton and University Hospital Southampton NHS Foundation Trust, Southampton, UK; 3 Southampton Education School, Faculty of Social Sciences, University of Southampton, Southampton, UK; 4 School of Computing, Engineering and Built Environment, Glasgow Caledonian University, Glasgow, UK; 5 Centre for Clinical and Community Applications of Health Psychology, University of Southampton, Southampton, UK; 6 Primary Care and Population Sciences, University of Southampton, Southampton, UK

**Keywords:** Adolescent, Health behaviours, Parents, Qualitative methods

## Abstract

**Objective:**

To identify the ways in which parental involvement can be incorporated into interventions to support adolescent health behaviour change.

**Design:**

Data from semi-structured interviews were analysed using inductive thematic analysis.

**Setting:**

Southampton, Hampshire, UK.

**Participants:**

A convenience sample of twenty-four parents of adolescents.

**Results:**

Parents consider themselves to play an important role in supporting their adolescents to make healthy choices. Parents saw themselves as gatekeepers of the household and as role models to their adolescents but recognised this could be both positive and negative in terms of health behaviours. Parents described the changing dynamics of the relationships they have with their adolescents because of increased adolescent autonomy. Parents stated that these changes altered their level of influence over adolescents’ health behaviours. Parents considered it important to promote independence in their adolescents; however, many described this as challenging because they believed their adolescents were likely to make unhealthy decisions if not given guidance. Parents reported difficulty in supporting adolescents in a way that was not viewed as forceful or pressuring.

**Conclusions:**

When designing adolescent health interventions that include parental components, researchers need to be aware of the disconnect between public health recommendations and the everyday reality for adolescents and their parents. Parental involvement in adolescent interventions could be helpful but needs to be done in a manner that is acceptable to both adolescents and parents. The findings of this study may be useful to inform interventions which need to consider the transitions and negotiations which are common in homes containing adolescents.

Adolescence is a period often characterised by poor health behaviours including high intake of energy-dense, nutrient-poor food and low levels of physical activity^(1)^. In the UK, many adolescents fail to meet Public Health England’s recommendations for a healthy diet and physical activity levels. Only 8 % of UK adolescents aged between 11 and 18 years eat five portions of fruit and vegetables a day^(2)^, and only 15 % of boys and 8 % of girls, aged 13–15 years, carry-out 1 h of moderate to vigorous activity per d^(3)^. Adolescents are the parents of future generations. Thus, intervening during adolescence to improve diet and physical activity has the potential for triple benefit: to the adolescent in the here and now, to the future health of the adolescent and to the health of the adolescent’s future offspring^(4,5)^. Although this age group has been identified as a priority group for health improvement, few interventions aiming to improve adolescent diet and physical activity levels show long-term effectiveness^(6–9)^.

Adolescence is a period of dramatic physical and psychosocial change^(10)^. Adolescents experience a desire for increased levels of autonomy over decisions in their life^(11)^. This new-found independence, coupled with increased exposure to factors outside of the family home, can lead to participation in health-compromising behaviours^(5)^. Nonetheless, parents still play an influential role in adolescents’ day-to-day lives even though the capacity for decision-making is increasing.

Health behaviour interventions targeting adolescents have been implemented using a number of different approaches with varying degrees of success^(6–8,12)^. It is argued that many of these health interventions are unsuccessful because they do not address adolescents’ desire to feel respected and fail to offer opportunities for adolescents to exercise autonomy over their health behaviours^(9,13,14)^.

Evidence indicates that interventions targeting children and adolescents which also involve parents have the potential to be successful, but results are varied^(7,8,15,16)^. Parents have been incorporated into interventions using multiple ‘indirect’ and ‘direct’ strategies^(15)^. Indirect parental engagement strategies have been more commonly used in intervention studies to date and often include delivering health information to parents via newsletters and web-platforms. Direct strategies have included parental attendance at educational and coaching sessions. Studies including such direct strategies are limited and may be subject to bias, as such selection bias where more highly motivated parents participate.

The important role parents play in their adolescents’ lives, if harnessed appropriately, could promote engagement with interventions outside of the immediate delivery setting. However, there is no clear consensus on what type of parental involvement is most effective^(15)^. In addition, little is known about parental views of the most acceptable ways of involving them in health interventions targeting their adolescents.

This study, therefore, adopted a qualitative approach to explore three research questions:

1. How do parents view their role in supporting their adolescents to eat healthily and be more physically active?
2. What factors influence the way in which parents choose to support their adolescents?
3. How can we help parents support their adolescents to make healthier choices?

## Methods

### Design

This exploratory qualitative study formed part of the developmental work for the Engaging Adolescents in CHanging Behaviour (EACH-B) study. The EACH-B study is a multi-component intervention aiming to support adolescents to engage in healthy dietary and physical activity behaviours^(17)^. The development of the EACH-B intervention was conducted using a Person-Based Approach which adopts user-centred methods to design and refine interventions. This involves in-depth qualitative interviews with stakeholders in order to ensure interventions are appropriate to the target population^(18)^. Reporting of this study follows COnsolidated criteria for REporting Qualitative research (COREQ) recommendations^(19)^.

### Study participants

A convenience sample of parents, who had at least one adolescent attending secondary school, was recruited from those who were part of an email list following their adolescent’s visit to LifeLab. Lifelab is an educational facility based at Southampton General Hospital, UK, and is primarily attended by students from Hampshire-based secondary schools between the ages of 11 and 18 years. Parents were sent an email explaining the details of the study. If they were happy to participate, they were asked to reply to the study email. Those who agreed were later contacted by a member of the research team to discuss the details of the study and organise a suitable time for the interview. Parents were excluded if they did not speak English or if they did not have an adolescent aged 11–18 years who was attending secondary school. Prior to participating in the interviews, all parents provided informed written consent.

### Setting

The study was conducted in 2018 in Southampton, a large city on the south coast of England, ranked the 67th most deprived of the 326 local authorities in England^(20)^. The interviews were conducted in locations that were convenient for the study participants, including places of work, home and over the telephone for the individual interviews. Group interviews were conducted at a hospital evening event for parents.

### Procedure

Four women researchers were involved in the interviews; S.S. (PhD student and research assistant) and S.T.S. (Post-doctoral research fellow) conducted the interviews. S.J. (MSc student and research assistant) and D. Watson (PhD student and research assistant) acted as observers in the interviews. All researchers received training in conducting qualitative interviews; S.S. and S.T.S. had previous experience of conducting qualitative research. The researchers were not known to the participants prior to correspondence about the study. Each participant completed a brief demographic questionnaire asking their age, gender, ethnicity and level of education. They were made aware that the research team was aiming to develop an intervention to improve diet and physical activity in adolescents. A semi-structured discussion guide was designed to explore parents’ views of their adolescent’s health and lifestyle, as well as factors which make it difficult for them to engage in healthy behaviours (Table 1). All interviews were audio-recorded and transcribed verbatim either by one of the authors (S.J.) or by a professional transcription company.

**Table 1:** Interview topic guide

Interview topic	Interview questions
Health	What does it mean to you to be healthy? What kinds of things do you do to keep healthy? How often do you and your adolescent talk about health and what it means to be healthy?
Parent life	What kinds of things do you do to keep healthy?
Family life	What is a typical day like for your family? How does your family decide what to eat at home? What kinds of things do you do with your adolescent to keep healthy? What kinds of things do you encourage your adolescent to do to keep healthy?
Your adolescent	What kinds of things does your adolescent like to eat? What kinds of activities does your adolescent engage in?
Barriers	What kinds of things make it difficult to keep healthy?
Support	What things in your life have helped you eat well and be active? What kind of things would help engage your adolescent in eating better? What kind of things would help encourage your adolescent to be more active?

### Analysis

**Fig. 1:**
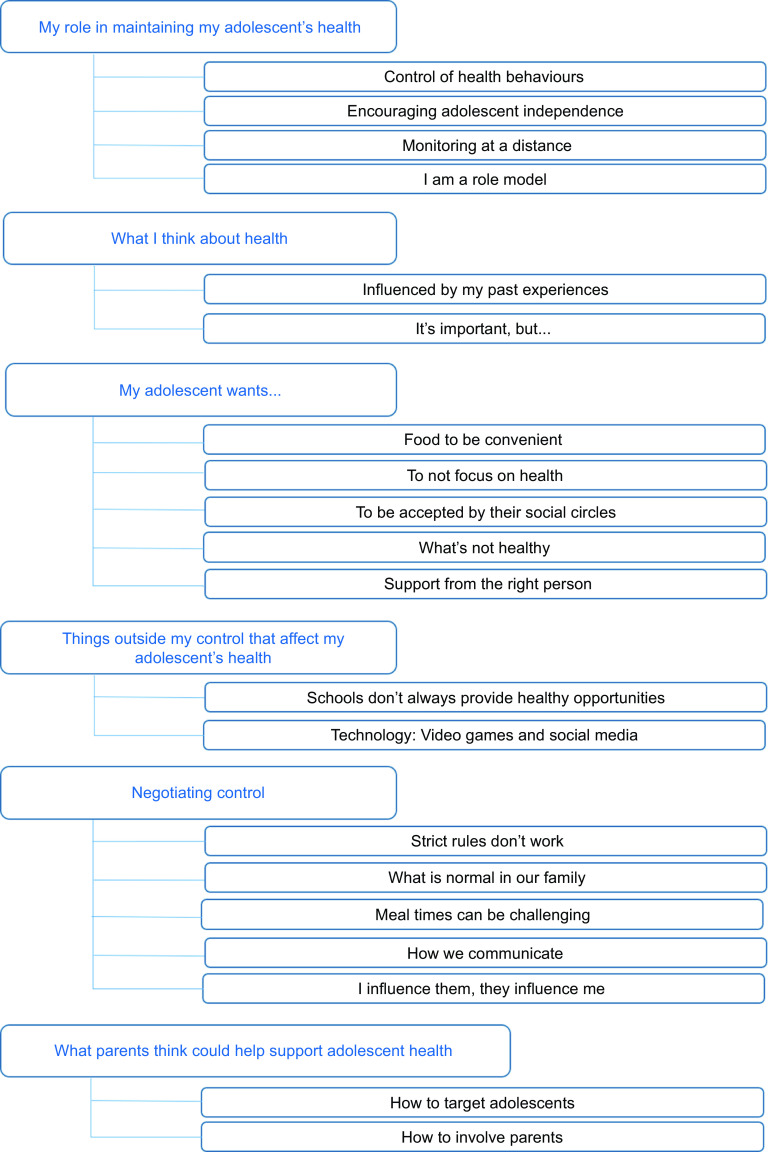
Coding frame used for thematic analysis

Interview recordings were analysed thematically using NVivo software (Version 12) (QSR International, version 12) to manage the data. Researchers familiarised themselves with the data by reading the interview transcripts and listening to the recordings. Inductive thematic analysis was conducted following established guidelines^(21)^. Three researchers (S.S., S.J., S.C.S.) worked independently to create initial codes in NVivo. After three transcripts were coded, the researchers met to discuss the similarities and differences between the codes. Codes were then organised into themes and sub-themes to create an initial coding frame. The coding frame was refined through coding of all transcripts (S.S., S.J., S.C.S.) until a sixth, final comprehensive coding frame was agreed (Fig. 1). Themes and sub-themes were compiled together with verbatim quotations and agreed with senior members of the research team (S.T.S., L.M., W.T.L., M.E.B.). This approach was conducted in line with a relativist ontological and subjective epistemic position, following the belief that reality is a matter of individual perspective and based on personal experience and insight^(22)^.

Researchers agreed that data saturation had been reached when no new topics arose from the final transcripts. Appropriate transcript excerpts were selected and agreed on by the researchers in order to accurately represent the meaning of each theme and sub-theme. The relationships between the themes were discussed by the research team and visualised by creating a thematic map.

## Results

A total of twenty-four parents participated in this study: eighteen in individual interviews and six in two focus group discussions, each consisting of three participants. Each interview lasted approximately 20–45 min. Six individual interviews were conducted via telephone; all other interviews were conducted face-to-face. All participants were women. The majority (71 %) were aged between 40 and 49 years and 96 % identified as white (Table 2).

**Table 2:** Characteristics of the parents

Characteristic	*n*	%
Age (years)		
**30–39	2	8
**40–49	17	71
**50–59	5	21
Gender		
**Women	24	100
**Men	0	0
Ethnicity		
**White	23	96
**Arabic	1	4
Highest qualification		
**Lower secondary education or below (GCSE)	2	8
**Upper secondary education (A-levels or equivalent)	7	29
**Post-secondary education (Higher National Diploma or equivalent)	2	8
**Bachelor’s degree or above	10	42
**Other	3	13

**Fig. 2:**
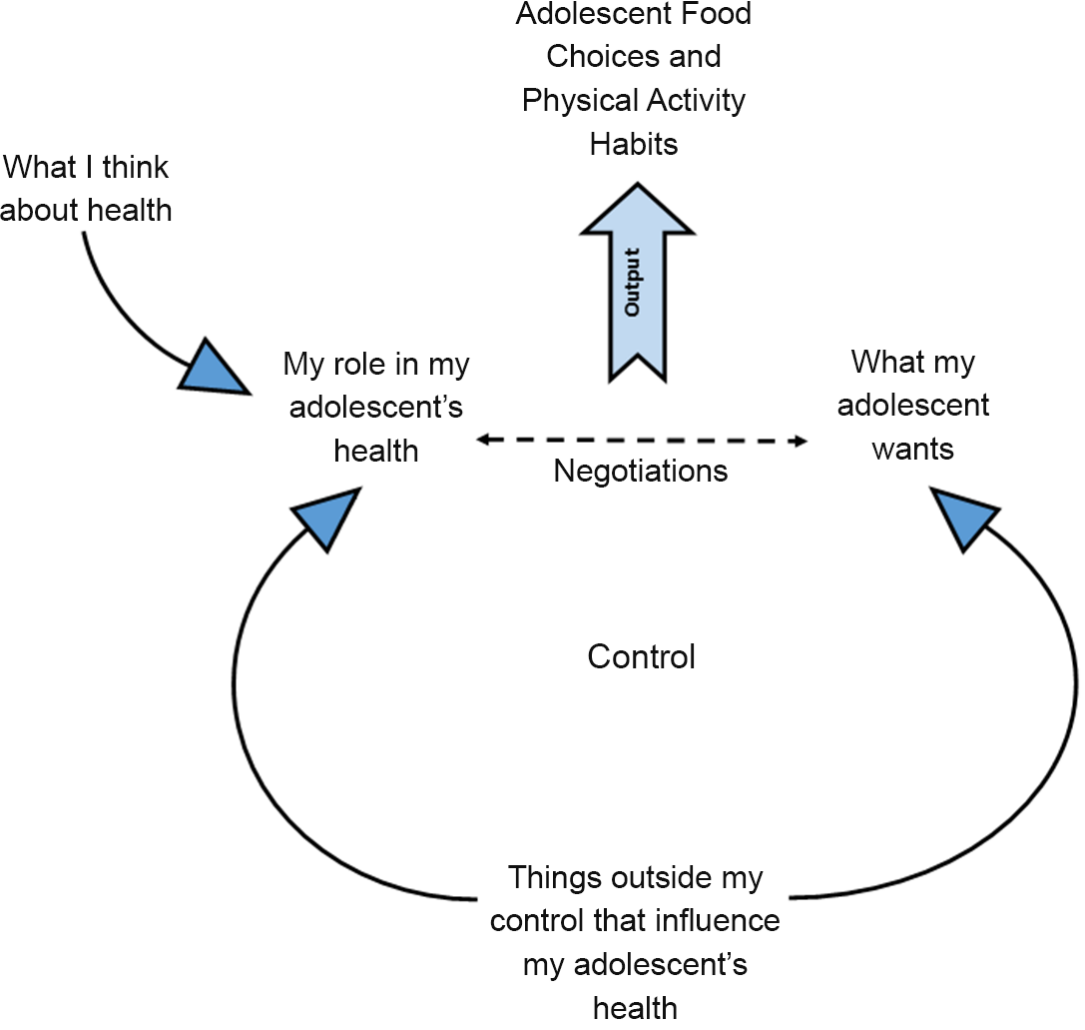
Thematic map showing parents’ perspectives of their role in supporting adolescent health

Six themes, consisting of multiple sub-themes, were identified by the analysis. Figure 1 shows the final coding frame which was used for the thematic analysis. Each theme is described alongside illustrative quotes from the interviews. Figure 2 shows the relationships between themes one to five and how they centre around the parents’ sense of control.

### Themes

#### My role in maintaining my adolescent’s heath

Parents described exerting authority over their households in several ways, including being in control of food preparation and rules for behaviour within the household.

*‘I am the one who controls the meals I guess, in the sense that I cook them, I put them on the table’- [Parent Group 1]*

Parents recognised that promoting independence and encouraging adolescents to make some of their own choices are important aspects of their parenting role.

*‘More recently I try and give my daughter a bit more control over her food, not because she’s requested it but I guess this comes from a kind of teaching background’- [Parent Interview 1]*

Even though parents viewed promoting independence in their adolescents as important, they described a reluctance to step back fully from their traditional parental role. They discussed ways in which they attempted to be attentive and supervised their adolescent whilst trying not to be directly involved. This involved checking their homework and food choices when out of the home using mobile apps and monitoring their social media use.

*‘I try to police it, he’s got like an Instagram account so I’ve set one up for me and I can follow him, so I know what he’s up to. He’s got a Facebook account and again I’m his friend on Facebook so again I can kind of see what he’s up to’- [Parent Interview 2]*

Parents described themselves as role models to their adolescents but acknowledged they did not always model healthy behaviours. They described modelling behaviours in relation to their own actions as well as the actions of other parents.

*‘If you’ve got inactive parents, your children aren’t gonna be active’- [Parent Interview 6]*

#### What I think about health

As depicted in Fig. 2, parents recognised that their personal perspective of health influenced how they viewed their role in promoting health in their adolescent. Many parents discussed their own or family members’ experiences of ill health and participation in healthy behaviours. They also outlined the ways in which past experiences influenced their current thoughts about health and leading a healthy lifestyle.

*‘So health is quite a complex thing in our family. We’ve had, well I’ve had, particular periods of extremely bad health, and so therefore being healthy has been something that we all take quite seriously in an attempt to counteract that as much as we possibly can.’- [Parent Interview 1]*

Parents not only described the importance of taking steps to ensure their families’ good health but also acknowledged that this is not always a priority for themselves or their adolescent.

*“What do you guys tend to do, to be healthy? - [Interviewer]*

*Honestly, we don’t do enough, we don’t do a lot.”- [Parent Group 2]*

*‘I personally think it’s really important, but it’s not just about eating. I think it’s about exercise as well, and doing them both.’- [Parent Interview 6]*

Parents described barriers to leading a healthy lifestyle and how these justified why healthy habits are not enforced in the family setting.

*‘You’ve also got to remember that, you know, some of them [other parents] are living on a very, very tight budget. It is cheaper to buy convenience food. So much easier to buy a packet of biscuits for, you know, something pence. Give them a couple of biscuits, done.’- [Parent Group 1]*

#### My adolescent wants…

Parents believed that an important factor in determining adolescents’ food choices was convenience and accessibility. They saw their adolescents as wanting foods to be prepared for them or for it to be very simple to prepare themselves. Parents believed that adolescents would not go out of their way to make healthy food choices but are open to healthy choices if they are available and convenient.

*“So, when he comes home from school, he definitely wants food. But he is happy for me to say to him, “Why don’t you have, cheese and biscuits?” rather than just having chocolate. I think he’s more interested in just having food, it’s not just that he wants to just eat chocolate or something. - [Parent Interview 13]*

Parents suggested adolescents were not motivated by health messages which are often perceived as boring. They described their adolescents as being willing to participate in healthy lifestyle behaviours as long as there was a reason for doing so that was not just health.

*‘In terms of sort of physical exercise, the dog has made a huge, huge difference to us as a family. We walk miles. Family holidays now involve walking a lot and even this week my son had a half day off school, it was beautiful like this and I said, “Would you come with me, with the dog?” And, he did come, and we enjoyed the sunshine, we had time together and walked the dog’- [Parent Interview 9]*

Parents emphasised that their adolescents would only engage with activities they enjoyed rather than those they classed as boring.

*‘They’ve gotta enjoy it. You’ve gotta find something they enjoy’– [Parent Interview 6]*

Parents recognised the pressure adolescents feel to be accepted and fit in with their peers, and how this can negatively affect their diet and exercise choices.

*‘Well my kids have said, and I can well believe this, that they do it to be with other kids that are buying food. And they don’t want to feel left out, that they’re not doing that kind of thing. So I think it’s peer pressure, to be going, to be getting that stuff.’ - [Parent Group 2]*

Conversely, friends and peers could also have a positive influence regarding being healthy and active.

*‘She’s got some friends who like running… I think parents have got them into cross country club and running clubs, and so in their little group that’s acceptable, ‘cause there’s some kids who do it. I just think it needs a couple of them to be brave and they can then set the norm’- [Parent Interview 5]*

Parents believed that when adolescents had autonomy over their health decisions, they made less healthy choices. They described their adolescent’s tendency to select unhealthy foods when they were not around to guide these decisions.

*‘When she first went to the senior school, I gave her money on her account … she immediately was buying doughnuts and all that kind of stuff.’- [Parent Interview 1]*

Parents also believed that their adolescent needed prompting to be physically active.

*‘If my son had the choice, he would much rather stay in front of a screen’ – [Parent Group 1]*

Parents recognised the importance of adolescents having the right individuals to provide support and encourage healthy behaviours. Parents specified that not everyone can fulfil these roles and that these people need to be acceptable to their adolescents.

*‘It [giving health advice] shouldn’t be done by someone at school. I mean it can be done by someone in the school, but not by a teacher who they wouldn’t respect.’- [Parent Interview 3]*

#### Things outside my control that affect my adolescent’s health

Figure 2 illustrates that parents recognised a number of factors that they considered to be outside their control that influenced what their adolescent wanted and how they viewed their role in promoting adolescent health. Parents perceived that schools did not always provide opportunities for their adolescents to participate in healthy behaviours but appeared to feel there was nothing they could do about it.

*‘She’s doing food tech at school and has learned to make a few meals, none of them particularly healthy, interestingly, you know chicken goujons, pizza, muffins, you know they’ve all been those sorts of things. I think its quick food, because they have such short lessons. They have to teach you know, it’s not nutrition they’re learning.’ - [Parent Interview 1]*

Parents were aware that technology such as smartphones and video games is valued by adolescents and felt that these got in the way of adolescents leading a healthy lifestyle.

*‘Her activity levels are low, way too low. And for me, it’s the phone. I have real, real issues with the mobile phone. Huge issues’- [Parent Group 3]*

*‘We had a complete break-down, because we were going on holiday and she was not going to have internet. ‘I’ve had a forty-day streak with somebody, I can’t.’’- [Parent Group 2]*

#### Negotiating control

Parents discussed the changing dynamics of control between them and their adolescent, resulting in a culture of compromise between the parent and adolescents which was influenced by several factors inside and outside the home (Fig. 2). Parents suggested that their adolescent would ignore or rebel against strict household rules, so they would sometimes relax or modify these in an attempt to maintain control and establish an acceptable compromise.

*‘If you’re too strict with them about what they can eat, then they’ll rebel.’ - [Parent Group 2]*

*‘I’ve learnt that the more you badger, the worse it gets’- [Parent Interview 10]*

As adolescents gained exposure to factors outside of the family unit, there could be increasing conflict between what is normal for the family and what is normal for others.

*‘I parent differently, I struggle with that all the way, you know, “my friends don’t have to…” Well, your friends don’t live in this house.’- [Parent Interview 11]*

Parents described changes in their adolescents’ eating habits which can make mealtimes challenging. To avoid conflict, they provided food they know will be acceptable even if this means cooking less healthy meals.

*‘I mean, there are times when he refuses things like pasta and rice… and sometimes I end up giving him chips.’ – [Parent Interview 13]*

Parents also described the challenges of talking to their adolescent about health behaviours.

*‘Your mum and dad saying, “Oh, this is what you ought to do,” it’s gonna be like, no. I don’t think they generally take very kindly… or a lot of them don’t.’- [Parent Interview 10]*

*‘I think the most important thing to us is just trying and keep talking with them and I guess as they get older that might get a little bit difficult. But for us, I think is trying to just keep explaining why we want them to do…you know, explaining the reasoning behind the things.’– [Parent Interview 18]*

Parents reported a reciprocal influence in their interactions with their adolescents. While parents could encourage adolescents to eat healthier and be more active, they could also be influenced by adolescents to pursue a healthier lifestyle themselves.

*“On the days when I haven’t gone for a run, they go, “Oh, hang on a minute, you’re telling me to go, but you haven’t been. Actually, we could do this together.”- [Parent Interview 8]*

#### What parents think could help support adolescent health

Parents stated that their adolescents appreciated rewards and thought that this could be a useful way to encourage engagement with healthy behaviours. Parents recognised that technology was important to their adolescents and that findings ways in which it could be used to benefit health was important.

*‘My daughter’s recently been doing virtual medals, where you run a 5 K or something, and then you claim your medal at the end of it, and it comes through the post.’- [Parent Group 2]*

Parents also highlighted that it was important to make participating in healthy behaviours appealing to their adolescents.

*‘It’s got to be a reason to engage in it in some way… it needs to be something that isn’t gonna come across as boring, or sort of telling you what to do.’- [Parent Interview 3]*

*‘I think for kids it needs to be really easy, and they need to be involved so much that they don’t want to miss it’- [Parent Interview 1]*

When asked how they would like to be involved in health interventions, some parents suggested that facilitating the link between the parent and the adolescent might help them find ways to support with healthy behaviour changes.

*‘You could set the challenge of the child creating a weekly menu and you could have the parent share that, in terms of the shopping list’ – [Parent Interview 5]*

Others stated that difficulties might arise when trying to engage parents with this sort of health intervention.

*‘I think a lot of parents think they know things and they probably wouldn’t bother attending seminars or things like that.’- [Parent Interview 6]*

Some highlighted that finding ways to involve parents that were acceptable to their adolescents might be challenging as they valued their privacy.

*‘[Name]’s on Instagram but I’ve no idea what her site is ‘cause that’s all blocked and hidden from us.’- [Parent Interview 14]*

## Discussion

This paper identified an overarching theme describing the changing dynamics between parents and adolescents which concern shifting perceptions of control. This was at the centre of how parents viewed their role in supporting their adolescents to eat healthily and be physically active. Parents recognised that their role in supporting these behaviours was reducing as their adolescents’ autonomy and independence increased. Parents described negotiations with their adolescents as playing a role in determining the healthiness of their adolescents’ food choices and activity levels. Parents found that their attempts to guide and advise their adolescents were poorly received and often caused a breakdown in communication.

Parents also recognised several external factors that presented barriers to healthy behaviours in their adolescents and these also influenced how they viewed their role in supporting healthy behaviours.

The parents we spoke to felt their adolescents lacked the ability to self-regulate their behaviours and, if left to their own devices, would not be physically active and would choose unhealthy food, especially in environments where such foods were readily available. They also believed that, unless healthy behaviours were normalised in their households from an early age, adolescents tended towards unhealthy behaviours such as eating foods high in fat, salt and sugar and low physical activity levels.

Parents recognised that interventions need to be appealing, to fit into their adolescents’ lives, and certainly not be viewed as boring. They highlighted that interventions focusing solely on health were unlikely to engage adolescents. Parents also stated that interventions containing parental components needed to fit into their lives otherwise they risked being side-lined as they will not be viewed as a priority in parents’ busy lives.

### Comparisons with previous literature

In line with the ‘negotiating control’ theme identified in this study, previous qualitative research exploring parent and adolescent attitudes towards sugar-sweetened beverage consumption and screen time has described regular disagreements between parents and adolescents about everyday decisions that influence the behaviours of the family in the household^(23)^. This research recognised that any adolescent interventions must acknowledge this dynamic in order to be effective^(23)^. Other qualitative work has also described the view held by parents that controlling the home food environment is one of the most effective methods of promoting healthy food choices by their adolescents^(23,24)^. However, evidence suggests that this practice may vary by socio-economic status, a factor that was not explored in the current study^(24)^. A limitation of previous research is that it has only focused on specific dietary behaviours such as sugar-sweetened beverage consumption, while the current study focused on exploring parents’ views of the more complex behaviour of overall food choice. Parents have also previously described a lack of control when it comes to countering the negative influence of peers on the health behaviours of their children and feeling lower levels of control over other household activities such as screen time^(23,25)^.

Other authors have identified open communication between parents and adolescents as an important tool for effectively supporting weight management interventions for obese and overweight adolescent populations^(25,26)^. Parents in the current study recognised that communication with their adolescent was often difficult and ‘the more they badgered’ their adolescent the less likely it was for their advice to be accepted. This suggests that training for parents in effective communication skills may be an important strategy to include in interventions for non-clinical, as well as clinical, populations. This is supported by other qualitative research with parents and adolescents that also proposes positive communication styles to be the most effective in promoting healthy behaviours^(23)^.

### Implications for public health

Parents described the changing dynamics of their relationships with their adolescents as a major influence on how they provided support, with negotiations seeming to be part of everyday life. Branje describes these negotiations as a reorganisation of the parent–adolescent relationship from one that is vertical, with the parent in a position of power, to one that is horizontal, where power is more equally balanced^(27)^. As part of these negotiations, parents appeared to value their role in promoting independence in their adolescents, though doubted that their adolescents would participate in healthy behaviours if left to their own devices. Previous research highlights that approaches to enhance autonomy are more effective in promoting healthy eating in children and adolescents than more controlling strategies^(28)^.

To date, the majority of parental components in adolescent health interventions have used indirect, but overt, methods to encourage healthy behaviours in adolescents. Such methods have focused on providing information to parents using newsletters, tip sheets and nutrition and physical activity information sheets^(15)^. Parents who took part in this study felt that they had less control over their adolescents’ food and physical activity choices than when they were children. These findings highlight the importance of how the information provided as part of these interventions is converted by parents into practical support for the adolescent, if at all. It is suggested that direct methods such as parent training and information sessions may be more effective than indirect methods^(15)^. However, parents themselves felt that such direct methods might be an ineffective way to engage other parents in interventions due to life pressures restricting their ability to attend such meetings. Participants reported that other parents might feel their lives were too busy and that some might feel that they had nothing to learn^(15)^.

Some parents in this study described how health behaviours, such as home cooking and participating in physical activity, had become normal in their families, having been established and practised for many years. Many parents felt that adolescents were open to eating healthy foods, at least at home, if they were convenient for the adolescent to access and eat. However, most parents still recognised significant challenges when attempting to encourage adolescents to consider swapping unhealthy behaviours for healthier choices. As described in the sub-theme *‘schools don’t always provide healthy options’,* parents viewed the food options that were often available on school premises as unhealthy and thought that adolescents would choice these options if they were available. Previous research has shown that the majority of food high in fat, salt and sugar which adolescents eat is consumed outside of the home^(29)^. Parents in this study perceived that they had very little control over their adolescents food choices outside the home. Adolescents are bombarded with advertising and promotions which encourage unhealthy food choices in these environments^(30)^. Public health interventions to encourage more healthful food environments are likely to play a role in promoting healthy dietary choices in adolescents when they are away from the home environment. Adolescent autonomy and sense of social justice have previously been incorporated into an experimental study to highlight the role of manipulative food marketing and promote healthier dietary choices^(31)^. Future health behaviour interventions may find incorporating similar techniques as a helpful way to raise adolescents awareness of unhealthy food environments and support them to be more critical of how environments shape their behaviours.

Future interventions should aim to equip parents with strategies to promote healthy autonomous behaviours. One such strategy may include facilitating effective communication between parents and adolescents^(32)^. Communication strategies which were not forceful or pressuring have been found to be preferred by both parents and adolescents^(33)^. This supports previous research which investigated the role of authoritarian and authoritative parenting styles in relation to adolescent eating behaviours. An authoritative parenting style, one that provides structured guidance and takes the views of the adolescent into consideration, has been shown to be positively associated with increased fruit and vegetable consumption and breakfast intake^(34)^. Qualitative research shows that adolescents do not respond positively to authoritarian parenting styles, characterised by strict enforcement of parental rules with little input from the adolescent, as adolescents reported feeling urge to rebel by eating unhealthy foods if they felt they were being lectured by their parents^(35)^. Parental communication that promotes autonomy in adolescents aligns with adolescents’ desire to feel respected and have the potential to increase the effectiveness of interventions^(9)^. One potential way of empowering parents with effective communication strategies could be through the delivery of Healthy Conversation Skills training. Healthy Conversation Skills offers a set of accessible, theory-based skills focusing on listening, reflecting and goal-setting^(36,37)^. The use of such techniques may enable adolescents to feel that their views are being listened to, and feel more independent, while still allowing parents to guide their adolescents to identify their own health goals and explore ways of achieving them that they consider acceptable and feasible^(36,37)^. Training in these skills may be more enticing to parents than information training about healthy lifestyle behaviours, as the focus can be placed on building and fostering relationships between parents and adolescents rather than only focusing on health. This training has not previously been provided to parents but has been shown to be effective in primary health care settings. Providing Healthy Conversation Skills training via a digital platform may be one way to overcome the need for face-to-face training with parents, which has been a barrier to intervention delivery in previous studies^(38)^.

### Strengths and limitations

The qualitative interviews in this study have provided rich data on parental perspectives of adolescents’ lives. This perspective has not often been considered when developing interventions that target adolescents. Despite the best efforts of the authors, it was not possible to recruit any fathers to participate in this study. This recruitment issue may be related to the increased parental involvement of mothers who often take on a traditional primary caregiver role within the family. The difficulty of recruiting fathers to participate in this type of research has been highlighted previously^(39,40)^. It is likely that fathers have a different perspective on their adolescents’ lives, and the benefit of including them in future studies would be significant. Most participants in this study were white (representative of locality) and were educated to upper secondary school standard or above. Interviews with more diverse groups of parents may have produced different data. The interpretation of the qualitative data presented in this paper is only one possible interpretation and will have been influenced by the experiences and beliefs of the research team. In order to ensure the research findings from this study fairly represented the views of the interviewees, a rigorous process was adopted that involved double-coding of data, with disagreements being resolved through team discussions. The final interpretation of the data was agreed by all team members after multiple discussions.

## Conclusions

This study found that parents recognise and value the importance of promoting good health behaviours in their adolescents but find doing so difficult due to the increasing lack of influence they have over elements of their adolescents’ lives. When designing adolescent health interventions that include parental components, researchers need to be aware of the disconnect between public health recommendations and the everyday reality for adolescents and their parents. This research may be useful to inform interventions which need to consider the transitions and negotiations which are common in homes containing adolescents. Future qualitative research using dyadic interviews, conducted with both parents and adolescents, may provide insight into the shared experiences of the changing levels of control in their lives and inform how, and when, to deliver a communication intervention. Researchers designing these health interventions need to recognise that a ‘one size fits all’ approach is unlikely to produce successful long-term health behaviour change.

## References

[1] Bates B , Cox L , Nicholson S et al. (2016) National Diet and Nutrition Survey Results from Years 5 and 6 (combined) of the Rolling Programme (2012/2013–2013/2014). London: PHE.

[2] Public Health England (2018) National Diet and Nutrition Survey Results from Years 7 and 8 (combined) of the Rolling Programme (2014/2015 to 2015/2016). London: PHE.

[3] Digital NHS (2016) Health Survey for England 2015 Physical Activity in Children. London: Digital NHS.

[4] Barker M , Dombrowski SU , Colbourn T et al. (2018) Intervention strategies to improve nutrition and health behaviours before conception. Lancet 391, 1853–1864. 29673875 10.1016/S0140-6736(18)30313-1 PMC6075694

[5] Patton GC , Sawyer SM , Santelli JS et al. (2016) Our future: a Lancet commission on adolescent health and wellbeing. Lancet 387, 2423–2478. 27174304 10.1016/S0140-6736(16)00579-1 PMC5832967

[6] Calvert S (2019) Delivering in-school interventions to improve dietary behaviours amongst 11- to 16-year-olds: a systematic review. Obes Rev 20, 543–553. 30550629 10.1111/obr.12797

[7] Rose T , Barker M , Maria Jacob C et al. (2017) A systematic review of digital interventions for improving the diet and physical activity behaviors of adolescents. J Adolesc Health: Offic Publ Soc Adolesc Med 61, 669–677. 10.1016/j.jadohealth.2017.05.024 PMC5702542 28822682

[8] Stice E (2006) A meta-analytic review of obesity prevention programs for children and adolescents: the skinny on interventions that work. Psychol Bull 132, 667–691. 16910747 10.1037/0033-2909.132.5.667 PMC1876697

[9] Yeager DS (2018) Why interventions to influence adolescent behavior often fail but could succeed. Perspect Psychol Sci: J Assoc Psychol Sci 13, 101–122. 10.1177/1745691617722620 PMC5758430 29232535

[10] World Health Organization (2014) Health for the World’s Adolescents. A Second Chance in the Second Decade. Geneva: WHO.

[11] Stangor C (2014) Adolescence: Developing Independence and Identity. Introduction to Psychology. Boston: FlatWorld.

[12] Chen JL & Wilkosz ME (2014) Efficacy of technology-based interventions for obesity prevention in adolescents: a systematic review. Adolesc Health Med Ther 5, 159–170. 25177158 10.2147/AHMT.S39969 PMC4132224

[13] Bassett R (2008) Autonomy and control: the co-construction of adolescent food choice. Appetite 50, 325–332. 17936413 10.1016/j.appet.2007.08.009

[14] Bryan CJ , Yeager DS , Hinojosa CP et al. (2016) Harnessing adolescent values to motivate healthier eating. Proc Natl Acad Sci 113, 10830. 27621440 10.1073/pnas.1604586113 PMC5047199

[15] Hingle MD , O’Connor TM , Dave JM et al. (2010) Parental involvement in interventions to improve child dietary intake: a systematic review. Prev Med 51, 103–111. 20462509 10.1016/j.ypmed.2010.04.014 PMC2906688

[16] Van Lippevelde W , Verloigne M , De Bourdeaudhuij I et al. (2012) Does parental involvement make a difference in school-based nutrition and physical activity interventions? A systematic review of randomized controlled trials. Int J Public Health 57, 673–678. 22301709 10.1007/s00038-012-0335-3

[17] ISRCTN Registry (2019) Engaging adolescents in changing behaviour: a programme of research to improve the diets and physical activity levels of adolescents. 10.1186/ISRCTN74109264 (accessed February 2020).

[18] Yardley L , Morrison L , Bradbury K et al. (2015) The person-based approach to intervention development: application to digital health-related behavior change interventions. J Med Internet Res 17, e30. 25639757 10.2196/jmir.4055 PMC4327440

[19] Tong A (2007) Consolidated criteria for reporting qualitative research (COREQ): a 32-item checklist for interviews and focus groups. Int J for Qual Health Care 19, 349–357. 10.1093/intqhc/mzm042 17872937

[20] Southampton City Council S (2015) Index of multiple deprivation (2015) analysis of overall changes since 2010. https://data.southampton.gov.uk/images/imd2015-analysis-of-changes-since-2010_tcm71-406229.pdf (accessed February 2021).

[21] Braun V & Clarke V (2006) Using thematic analysis in psychology. Qual Res Psychol 3, 77–101.

[22] Braun V & Clarke V (2013) Successful Qualitative Research. A Practical Guide for Beginners. London: SAGE.

[23] Hattersley LA , Shrewsbury VA , King LA et al. (2009) Adolescent-parent interactions, attitudes around screen time, sugary drink consumption: a qualitative study. Int J Behav Nutr Phys Activity 6, 61. 10.1186/1479-5868-6-61 PMC2747835 19740410

[24] Roth-Yousey L (2012) A qualitative study to explore how parental expectations and rules influence beverage choices in early adolescence. J Nutr Educ Behav 44, 644–652. 22236494 10.1016/j.jneb.2011.07.005

[25] Azar KMJ , Halley M , Lv N et al. (2020) Differing views regarding diet and physical activity: adolescents versus parents’ perspectives. BMC Pediatr 20, 137–137. 32220230 10.1186/s12887-020-02038-4 PMC7099828

[26] Hadley W , McCullough MB , Rancourt D et al. (2015) Shaking up the system: the role of change in maternal-adolescent communication quality and adolescent weight loss. J Pediatr Psychol 40, 121–131. 25214645 10.1093/jpepsy/jsu073 PMC4288305

[27] Branje S (2018) Development of parent–adolescent relationships: conflict interactions as a mechanism of change. Child Dev Perspect 12, 171–176.

[28] Baumrind D (2005) Patterns of parental authority and adolescent autonomy. New Dir Child Adolesc Dev 2005, 61–69. 10.1002/cd.128 16121897

[29] Toumpakari Z (2016) Adolescents’ non-core food intake: a description of what, where and with whom adolescents consume non-core foods. Public Health Nutr 19, 1645–1653. 26878965 10.1017/S1368980016000124 PMC10270939

[30] Truman E & Elliott C (2019) Identifying food marketing to teenagers: a scoping review. Int J Behav Nutr Physical Activity 16, 67. 10.1186/s12966-019-0833-2 PMC6700978 31426809

[31] Bryan CJ (2019) A values-alignment intervention protects adolescents from the effects of food marketing. Nat Hum Behav 3, 596–603. 30988478 10.1038/s41562-019-0586-6 PMC6784541

[32] Tu AW , Watts AW , Chanoine JP et al. (2017) Does parental and adolescent participation in an e-health lifestyle modification intervention improves weight outcomes? BMC Public Health 17, 352. 28438202 10.1186/s12889-017-4220-0 PMC5402679

[33] Shrewsbury VA , King LA , Hattersley LA et al. (2010) Adolescent-parent interactions, communication preferences regarding body weight, weight management: a qualitative study. Int J Behav Nutr Physical Activity 7, 16. 10.1186/1479-5868-7-16 PMC2831813 20205918

[34] Sleddens EFC , Gerards S , Thijs C et al. (2011) General parenting, childhood overweight and obesity-inducing behaviors: a review. Int J Pediatr Obes 6, E12–E27. 21657834 10.3109/17477166.2011.566339

[35] Krølner R , Rasmussen M , Brug J et al. (2011) Determinants of fruit and vegetable consumption among children and adolescents: a review of the literature. Part II: qualitative studies. Int J Behav Nutr Physical Activity 8, 1–38. 10.1186/1479-5868-8-112 PMC3260149 21999291

[36] Barker M , Baird J , Lawrence W et al. (2011) The Southampton initiative for health: a complex intervention to improve the diets and increase the physical activity levels of women from disadvantaged communities. J Health Psychol 16, 178–191. 20709878 10.1177/1359105310371397 PMC3685267

[37] Lawrence W , Black C , Tinati T et al. (2016) ‘Making every contact count’: evaluation of the impact of an intervention to train health and social care practitioners in skills to support health behaviour change. J Health Psychol 21, 138–151. 24713156 10.1177/1359105314523304 PMC4678584

[38] Hammersley ML (2016) Parent-focused childhood and adolescent overweight and obesity eHealth interventions: a systematic review and meta-analysis. J Med Internet Res 18, e203. 27443862 10.2196/jmir.5893 PMC4974451

[39] Davison KK , Gicevic S , Aftosmes-Tobio A et al. (2016) Fathers’ representation in observational studies on parenting and childhood obesity: a systematic review and content analysis. Am J Public Health 106, e14–e21. 10.2105/AJPH.2016.303391 PMC5055776 27631735

[40] Panter-Brick C , Burgess A , Eggerman M et al. (2014) Practitioner review: engaging fathers--recommendations for a game change in parenting interventions based on a systematic review of the global evidence. J Child Psychol Psychiatr 55, 1187–1212. 10.1111/jcpp.12280 PMC4277854 24980187

